# Endophytic Fungi as Potential Biocontrol Agents against *Rhizoctonia solani* J.G. Kühn, the Causal Agent of Rice Sheath Blight Disease

**DOI:** 10.3390/biology11091282

**Published:** 2022-08-29

**Authors:** Mohammad Reza Safari Motlagh, Bahar Jahangiri, Dariusz Kulus, Alicja Tymoszuk, Behzad Kaviani

**Affiliations:** 1 Department of Plant Protection, Faculty of Agriculture, Rasht Branch, Islamic Azad University, Rasht 4147654919, Iran; 2 Department of Plant Protection, Faculty of Agriculture, Mehrgan Institute for High Education, Mahallat 3781654363, Iran; 3 Laboratory of Ornamental Plants and Vegetable Crops, Faculty of Agriculture and Biotechnology, Bydgoszcz University of Science and Technology, Bernardyńska 6, 85-029 Bydgoszcz, Poland; 4 Department of Horticultural Science, Rasht Branch, Islamic Azad University, Rasht 4147654919, Iran

**Keywords:** *Aspergillus fumigatus*, biological control, dual culture, *Oryza sativa*, *Trichoderma virens*, volatile metabolites

## Abstract

**Simple Summary:**

Rice, together with wheat and corn, is among the most important food crops for mankind. Half of the world’s population consumes rice, mainly in Asia, southern Europe, tropical America, and parts of Africa. Rice sheath blight, caused by *Rhizoctonia solani*, is one of the main diseases in rice production. The control of this pathogen is difficult due to its ecological behavior, broad host range, and the high survival rate of sclerotia under various environmental conditions. In this research, after morphological and molecular identification of fungal isolates, five superior isolates, including *Trichoderma virens*, *Trichoderma harzianum*, *Curvularia lunata*, *Aspergillus fumigatus*, and *Aspergillus awamori* were studied in the in vitro and greenhouse trials, towards their potential to inhibit *R. solani*. The statistical analysis showed a significant difference between the effectiveness of fungi used in the volatile metabolites assay and in terms of height and fresh weight of plants in the greenhouse. It can be concluded that *T. virens* and *A. fumigatus* are the most effective antagonists in rice sheath blight disease control.

**Abstract:**

The rice sheath blight disease, caused by *Rhizoctonia solani* J.G. Kühn fungus, is a major disease of *Oryza sativa* L. occurring all over the world. Therefore, efforts need to be undertaken to limit the spread of this pathogen, preferably by using environmentally friendly methods. In the present study, 57 fungal isolates were recovered by surface sterilization technique from 120 rice samples collected from paddy fields in Guilan province, Iran. Biological characterizations of the isolated taxa were performed in vitro, in the dual culture, volatile metabolites, and slide culture methods. Among the studied isolates, *Trichoderma virens* (J. H. Miller, Giddens and A. A. Foster) Arx was most effective in inhibiting the mycelial growth of *R. solani* in the dual culture (44.16% inhibition level), while *Aspergillus fumigatus* Fresen and *T. virens* had a 62.50–68.75% inhibition efficiency by volatile metabolites. In the slide culture, all of the isolates, except for *T. harzianum* Rifai, were effective in inhibiting the hyphae growth of *R. solani*. Under greenhouse conditions, rice plants inoculated with these potential antagonistic fungi showed a reduction in disease severity by even 41.4% as in the case of *T. virens*. Moreover, phenotypic properties of rice, such as plant height, fresh weight, and dry weight were increased in the plants inoculated with all antagonistic fungi tested, compared to the infected plants, except for the fresh weight of plants inoculated with *Curnularia lunata* (Wakker) Boedijn. The present in vivo and in vitro studies revealed that *T. virens* and *A. fumigatus* are the most effective antagonists in rice sheath blight disease control and could be applied in agricultural practice.

## 1. Introduction

Sheath blight caused by *Rhizoctonia solani* J.G. Kühn fungus is the second most detrimental rice disease [1]. As a soil-borne necrotrophic fungus that attacks many agricultural and non-agricultural plants, *R. solani* is one of the most widely studied taxa of *Rhizoctonia* genus due to the huge economic losses that it imposes and its broad range of hosts [2]. It is reported that rice sheath blight disease causes yield losses of 10–30% in the tropical regions of Asia [3]. In major rice-growing countries, the losses caused by this pathogen are significantly higher, sometimes reaching even 50% [3]. In Iran, sheath blight is the second most severe disease of rice, following the rice blast [4]. Traditional methods for dealing with this pathogen are known, however, and the risks of using chemical pesticides are evident. Efforts to find alternatives to chemicals have led to significant advances in the area of ecologically friendly methods of plant protection. The biocontrol of plant pathogens with endophytes is an inexpensive and eco-green technique with lasting effects and can provide a suitable alternative to chemicals [5].

Atanasova et al. [6] demonstrate that the initial *Trichoderma* mycotrophy has differentiated into several alternative ecological strategies ranging from parasitism to predation and saprotrophy. It provides first insights into the mechanisms of interactions between *Trichoderma* and other fungi that may be exploited for further development of bio-fungicides. Cook and Baker [7] studied 77 isolates of *Trichoderma* against several pathogens, including *R. solani*. According to the results of their dual culture test, 85% of the isolates were antagonists of *R. solani*. They also used soil- and irrigation-water-borne fungi from paddy fields against *R. solani* and found that *T. viride* and *Aspergillus niger* Tiegh. had the greatest effect in preventing the linear growth of this pathogen.

Gams and Bissett [8] found that *T. atroviride* P. Karsten inhibits the mycelial growth of *R. solani* and prevents the formation of its sclerotia. Their microscopic studies showed that over 25% of the pathogen mycelia were lysed close to the inhibiting halo and that most hyphal tips became bulb-like terminal enlargements. The addition of this antagonist to the soil in the greenhouse alleviated the severity of rice sheath blight. They also studied the biological control of *R. solani* by 16 different *Trichoderma* isolates in a dual culture test and four of the isolates were perfectly capable of preventing the growth of the pathogen.

Benitez et al. [9] isolated tricholine (a ribosomal inactivating protein) from *T. virens* (J. H. Miller, Giddens and A. A. Foster) Arx liquid culture medium and found that it had antifungal properties against *R. solani* by delaying its growth, absorption of amino acids, and protein biosynthesis. Padasht Dehkaee et al. [10] studied different taxa of *Trichoderma* and their potential to inhibit the growth of *R. solani* in vitro and reduce the disease severity under greenhouse conditions. They found that *T. viride* significantly reduced the mycelial growth and the number of pathogen sclerotia, and led to a reduction in the severity of sheath blight disease. Nzojiyobiri et al. [11] studied the effectiveness of *T. viride* and *T. harzianum* in reducing the severity of rice sheath blight disease on farms. These antagonists were applied as grain treatment, by spraying or soaking seedling roots in the spore suspension. The disease rating was reduced by 58% by using *T. viride*.

In vitro studies performed by Harman [12] showed that *T. viride* inhibited the development of *R. solani* and completely prevented the germination of its sclerotial bodies. He mentioned that the disease severity was only slightly reduced when these antagonists were added to the soil along with the pathogen, but the disease symptoms fully subsided when the spore suspension of *T. viride* was sprayed onto the aerial shoot of rice plants. Singh and Joshi [13] conducted in vitro and greenhouse examinations to determine the activity of *T. viride*, *T. koningii*, and *Gliocladium virens* against *R. solani*. They found that *T. koningii* Oudem. had the greatest effect in alleviating the sheath blight disease, and although *T. viride* showed good control potential in the dual culture test, its greenhouse performance was very poor. In the biological control of *R. solani* by using *Trichoderma* species, Naeimi et al. [14] concluded that seven strains of *T. harzianum*, *T. virens*, and *T. atroviride* had excellent performance in the glasshouse—even better than the fungicide propiconazole.

Kotasthane et al. [15] studied the biological control of *R. solani* as the cause of the root and collar rot of bean and found that various species of *Trichoderma* could inhibit the mycelial growth of this pathogenic fungus. Likewise, Anees et al. [16] introduced *Trichoderma gamsii* Samuels and Druzhinina as the most effective antagonist in the biological control of *R. solani*. In another study on the biological control of *R. solani* as the cause of bean root rot, *T. harzianum*, *Glomus intraradices* N.C. Schenck and G.S. Sm., and *Azotobacter chroococcum* Beijerinck were found to significantly reduce the disease severity, and their combined effect was greater than their individual effects [17]. Khodaee and Hemati [18] assessed the efficacy of *Trichoderma* isolates for the biological control of *Rhizoctonia* root rot of beans in Iran and concluded that mycoparasitism was the main mechanism used by *Trichoderma* isolates in their antagonistic activity. On the other hand, little is known about the use of biological control and endophytes other than *Bacillus* sp. strains in the stable prevention of the rice sheath blight disease [19,20,21], particularly in provinces of Iran.

Endophytic fungi (EFs) are a group of host-associated fungal communities that colonize the intercellular or intracellular spaces of plant tissues, providing multidimensional beneficial effects to their hosts while also gaining advantages. EFs have attracted much research interest as they can promote plant growth, act as biological control agents, or activate plant systemic resistances to (a)biotic stress [22]. The present research aimed to study the strategies of biological control of *Rhizoctonia solani*, the causal agent of rice sheath blight disease under in vitro and greenhouse conditions by various endophytic fungi, and examine their effects on the phenotypic traits of rice plants.

## 2. Materials and Methods

### 2.1. Sampling and Isolation

Diseased and healthy leaves of *Oryza sativa* L. ‘Hashemi’ were collected in sterile plastic bags and then transferred in an ice box to the laboratory for further work from Guilan province, Iran. Explants (pieces of stems and leaves) were surface sterilized with 0.5% (*v/v*) sodium hypochlorite solution, washed with sterile distilled water, and placed on the potato dextrose agar (PDA) (Merck Millipore, Burlington, MA, USA) in Petri dishes. The cultures were incubated at 28 °C in a 12/12-h day/night photoperiod for 6–15 days. Then, hyphal tipping or a single spore of the isolates of the recovered fungi were maintained on the half-strength PDA slants in test tubes as stock cultures [23]. 

### 2.2. Identification of Fungi

Morphological studies were performed in a water agar (WA) medium. Cuts of colonies were placed in a PDA medium for 2–3 days. Then, sections of the colonies were transferred to the WA medium for 7–30 days in an incubator at 27 °C and 12-h photoperiod. Afterward, morphological observations were taken based on colony, conidium, and conidiophore morphology, as well as other morphological traits [8,24,25,26,27,28]. Following the morphological identification of the fungal isolates, their molecular identification was performed by Bioneer, Daejeon, South Korea. The fungal isolates were cultured in the PDA medium. At the end of the incubation period and after the full growth of the samples, their genomic DNAs were extracted with the kit of CinnaGen (Tehran, Iran). For the molecular identification of the fungal isolates, the genomic ITS-rDNA region was assessed and the PCR was performed with a T-100 thermocycler (Bio-Rad, Hercules, CA, USA) to amplify the ITS-rDNA region [29]. The direct and reverse primer pairs required for the reaction were designed using the NCBI gene database and the Primer3 software package [30] and synthesized by Sinaclon, Tehran, Iran (Table 1). The initial PCR reaction was performed at a final volume of 25 µL using a PCR master mix by CinnaGen. The PCR thermal profile was set following White et al. [29] for amplifying the ITS-rDNA genomic region, and the electrophoresis was performed in the 1.5% (*w/v*) agarose gel (Sigma-Aldrich, St. Louis, MO, USA) at 120 V for 2 h. The electrophoretic separation was repeated twice. The PCR products of the studied samples were sequenced by Sinaclone (Tehran, Iran), and the similarity of the target sequence with the available sequences was assessed by the NCBI database using the BLAST algorithm [31]. The data from the sequence similarity analysis were phylogenetically arrayed by the neighbor-joining (NJ) method.

### 2.3. Biological Control Studies

A total of 20 endophytic fungal isolates were selected for the three biological control assays against *R. solani*, i.e., the dual culture, volatile metabolites, and slide culture tests.

#### 2.3.1. Dual Culture Method 

A mycelial disc, 5-mm-in-diameter, was taken from the margins of the 5–7-day-old culture of *R. solani* and transferred in a sterile hood to an 8-cm Petri dish containing a PDA medium at 2 cm from the margin of the plate. The cultures were incubated at 26 °C for 48 h. Then, a 5-mm-in-diameter mycelial disc from the studied antagonistic fungal isolates was transferred from the margins of the 5–7-day-old fungus (depending on the growth rate of the fungal colony) and placed at a 3 cm distance from the pathogen. The dual cultures were incubated at 26 °C, and the measurements were recorded 7–10 days later [32]. The control treatment consisted of the same design without the antagonistic fungi. After the incubation period, the diameter growth of *R. solani* was measured in the control and other experimental objects. The reduction in diameter growth was measured and compared to the control [32]. The reduction in the radial growth versus the control was measured by:

(1) $$\mathrm{Mycelial}\;\mathrm{growth}\;\mathrm{inhibition}\;\left(\%\right)=\frac{C\;-\;T}{C}\;\times \;100$$

in which C denotes the radial growth of *R. solani* in the control Petri dishes and T denotes the radial growth of the pathogen in the presence of the studied fungi [32].

#### 2.3.2. Volatile Metabolites Method 

A mycelial disc, 5-mm-in-diameter, from a 3-day-old culture margin of *R. solani* was placed in the center of a PDA Petri plate. Forty-eight hours later, a disc with a diameter of 5 mm from the 3-day-old culture of the candidate antagonistic fungi was placed in the center of a fresh PDA Petri plate. Then, the caps of these Petri dishes were removed in a sterile hood, and the dish containing *R. solani* was placed upside-down on the Petri dish containing the studied fungi. In the control object, the studied antagonistic fungi were replaced by a disc from the PDA medium. The inhibition percentage was calculated 10 days later, based on the formula mentioned in the dual culture method [32]. 

#### 2.3.3. Slide Culture Method

A laboratory slide was placed inside a 12-cm Petri dish on two L-shaped glass bars and sterilized. Then, molten 2% (*w/v*) water agar culture medium was poured on the slide to form a thin layer of agar. Small mycelial discs of the antagonistic fungus and *R. solani* were placed on the slide with 2-cm spacing. Approximately 3 milliliters of sterilized distilled water were added to each Petri dish to avoid their drying. The Petri dishes were kept at 26 °C. When the mycelia of the fungi reached each other, the slides were observed with an optical microscope and their interactions were investigated [32].

The effectiveness of the selected isolates in the inhibition of the mycelial growth of *R. solani* was evaluated twice.

### 2.4. Greenhouse Studies

The seeds of *Oryza sativa* L. ‘Hashemi’ were disinfected for one hour in a 30% (*v/v*) Clorox solution and then rinsed with sterile distilled water. A total of 45 plastic pots with a diameter of 11 cm were filled with paddy field soil (clay soil with low acidic pH). Ten seeds were planted in each pot. The pots were watered and placed in a greenhouse. The greenhouse temperature fluctuated between 27 °C and 34 °C in the daytime and 17–20 °C at night during the seedling growth period, and the air relative humidity was 80–100%. The seedlings were exposed to sunlight from cultivation until the 4–6-leaf stage. The plants were routinely watered to keep the pots constantly waterlogged. First, distilled water was manually sprayed on all the seedlings. Then, the spore suspensions of the pathogen and each of the antagonists were prepared and sprayed on the plants. The conidia were counted with a hemocytometer. A suspension containing 2.5 × 10^5^ mL^−1^ of conidia was used to inoculate the antagonistic fungi and, then, a suspension containing 4 × 10^5^ hyphal fragments per mL of sterile distilled water was used for inoculating *R. solani* (since this fungus is in the group of sterile fungi and does not produce conidia, its hyphal fragments were used). The surface adsorption of the plant was increased by using Tween-20 in a 1% (*v/v*) proportion [33]. The Horsfall–Barratt scale was used to measure the disease severity [34]. After 10 days, the disease rating was quantified by:

$$\mathrm{Disease}\;\mathrm{severity}=\frac{\;(N_{1}\;\times \;1)+(N_{2}\;\times \;2)+\ldots \;(N_{t}\;\times \;t)}{N_{1}+N_{2}+\ldots \;N_{t}}$$

where N represents the number of leaves at each degree of disease severity [35] and, then, the plant traits; such as height, fresh and dry weight; were measured. The effectiveness of the selected isolates in controlling rice sheath blight disease was evaluated twice. Two controls were included in this stage of the experiment—negative control (plants sprayed with sterile water) and positive control (plants inoculated with the pathogen only).

### 2.5. Data Analysis

The experiments were conducted in a completely randomized design with five treatments (antagonistic fungal species) and three replications. The analysis of variance (ANOVA) and the comparison of mean values, using the Least Significant Difference test (LSD) at *p* ≤ 0.01, were conducted in the SPSS software package (IBM, Armonk, NY, USA). The diagrams were plotted in the MS-Excel software package.

## 3. Results

**Figure 1:**
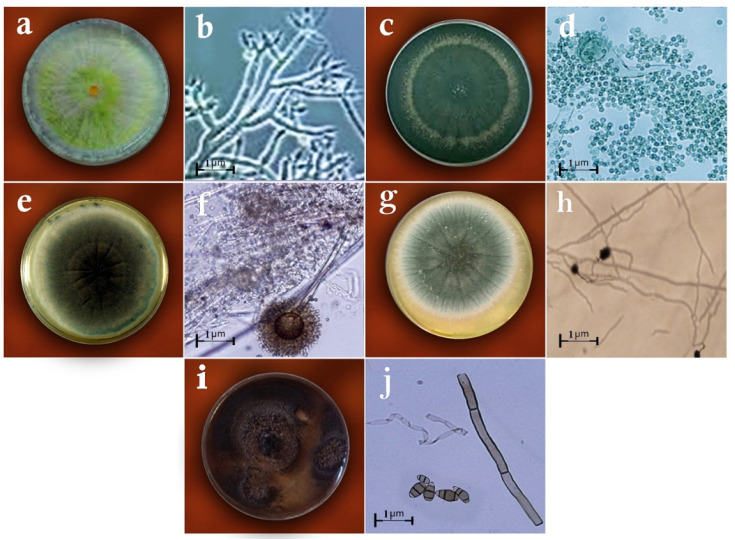
Antagonistic fungi used in the study: (**a**) the colony of *Trichoderma harzianum* on the PDA medium, (**b**) the conidia and conidiophores of *T. harzianum*, (**c**) the colony of *T. virens* on PDA, (**d**) the conidia and conidiophores of *T. virens*, (**e**) the colony of *Aspergillus* *fumigatus* on PDA, (**f**) the conidia and conidiophores of *Aspergillus* *fumigatus*, (**g**) the colony of *A. awamori* on PDA, (**h**) the conidia and conidiophores of *A. awamori*, (**i**) the colony of *Curvularia lunata* on PDA, (**j**) the conidia and conidiophores of *Curvularia lunata*.

**Figure 2:**
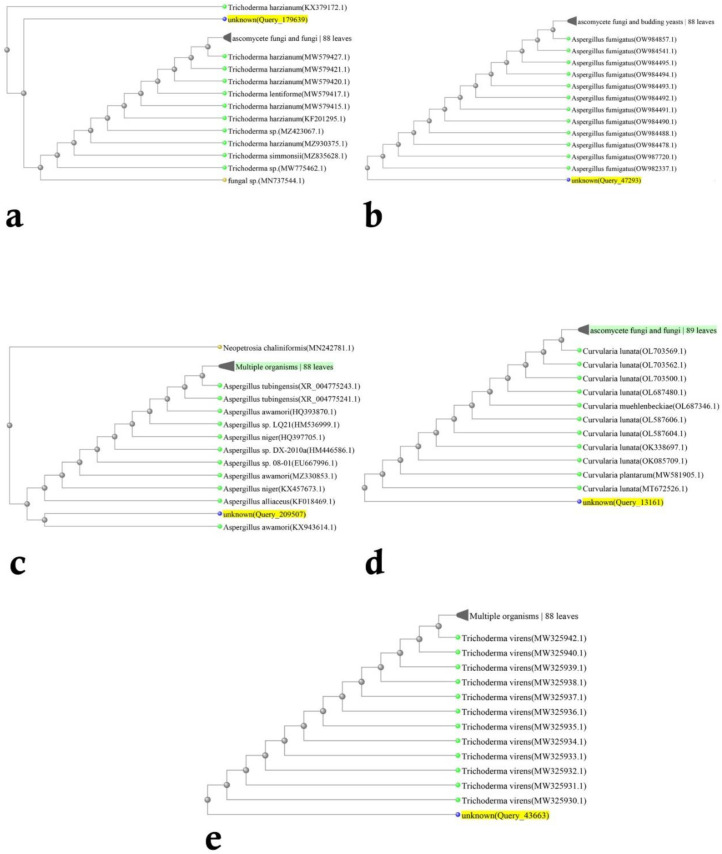
Phylogenetic trees resulting from the ITS-rDNA sequence analysis by the neighbor-joining method represent the location of the searched isolate in the vicinity of the cluster related to (**a**) *Trichoderma harzianum*, (**b**) *Aspergillus fumigatus*, (**c**) *Aspergillus awamori*, (**d**) *Curvularia lunata*, (**e**) *Trichoderma virens*.

**Figure 3:**
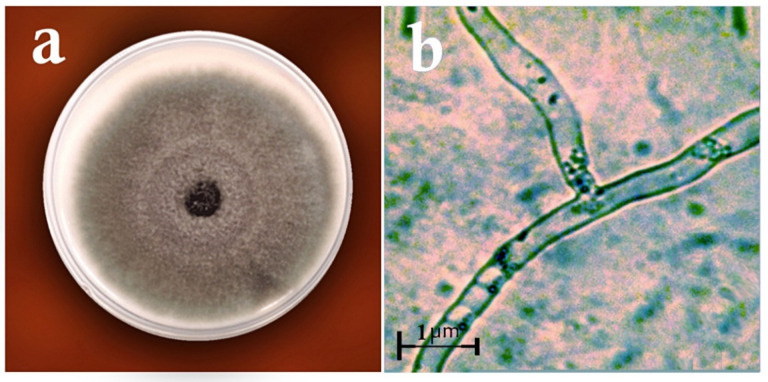
Pathogenic fungus used in the study: (**a**) the colony of *Rhizoctinia solani* on the PDA medium; (**b**) the hyphae of *R. solani*.

Based on the morphological and molecular characteristics, five fungal groups were identified. They included *Trichoderma harzianum* Rifai (MW579427), *Trichoderma virens* (J. H. Miller, Giddens and A. A. Foster) Arx (MW325930), *Aspergillus fumigatus* Fresen (OW982337), *Aspergillus awamori* Nakaz. (KX943614), and *Curvularia lunata* (Wakker) Boedijn (MT672526) (Figure 1 and Figure 2). In total, 20 fungal isolates were selected for the biological control assays in the laboratory (four isolates of each fungus species). From these isolates, five isolates were used in the greenhouse trials. On the other hand, the pathogenic fungal isolates belonged to the species *Rhizoctonia solani* J.G. Kühn (Figure 3). The pathogenicity of the identified strains (n = 33) was confirmed according to Koch’s postulates.

### 3.1. Dual-Culture Assay

**Figure 4:**
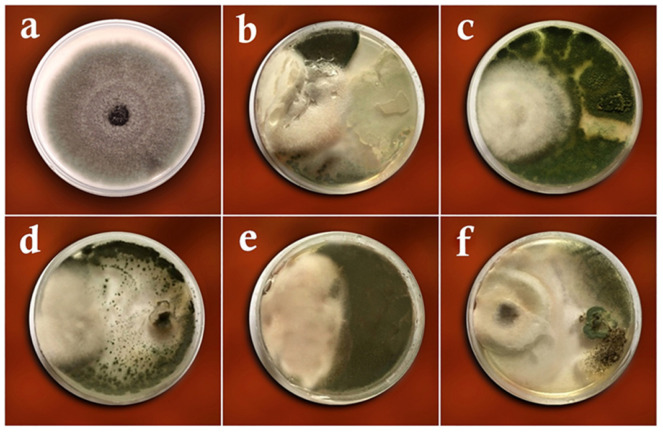
Dual culture test of *Rhizoctinia solani* versus the studied antagonistic fungi on the PDA medium: (**a**) *Rhizoctonia solani* only (control), (**b**) *Trichoderma harzianum*, (**c**) *Trichoderma virens*, (**d**) *Aspergillus fumigatus*, (**e**) *Curvularia lunata*, (**f**) *Aspergillus awamori*.

Table 2 presents the mean growth of *R. solani* isolates in the treatments with endophytic fungi and controls. The ANOVA showed no significant differences in the colonial growth and percentage of pathogen inhibition between the studied isolates in the dual culture test, which ranged from 37.61% (with *A. awamori*) to 44.16% (*T. virens*) (Table 2 and Figure 4). 

### 3.2. Volatile Metabolites Assay

**Figure 5:**
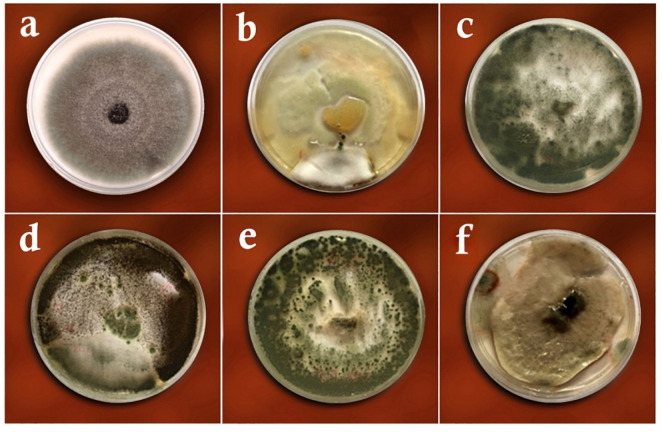
Volatile metabolites test of *Rhizoctinia solani* versus the studied antagonistic fungi on the PDA medium: (**a**) *Rhizoctonia solani* only (control), (**b**) *Trichoderma harzianum*, (**c**) *Trichoderma virens*, (**d**) *Aspergillus awamori*, (**e**) *Aspergillus fumigatus*, (**f**) *Curvularia lunata*.

The mean growth of *R. solani* isolates in the absence and presence of antagonistic fungal isolates in the volatile metabolite method is presented in Table 2. The statistical analysis revealed significant differences between the studied fungi in their effectiveness to inhibit the pathogens’ growth. The highest growth inhibition percentage was found with *A. fumigatus* and *T. virens* (62.50–68.75%). On the other hand, the lowest inhibition percentage was reported with *C. lunata* (38.46%), which differed significantly from all the other treatments (Table 2 and Figure 5).

### 3.3. Slide Culture Assay

**Figure 6:**
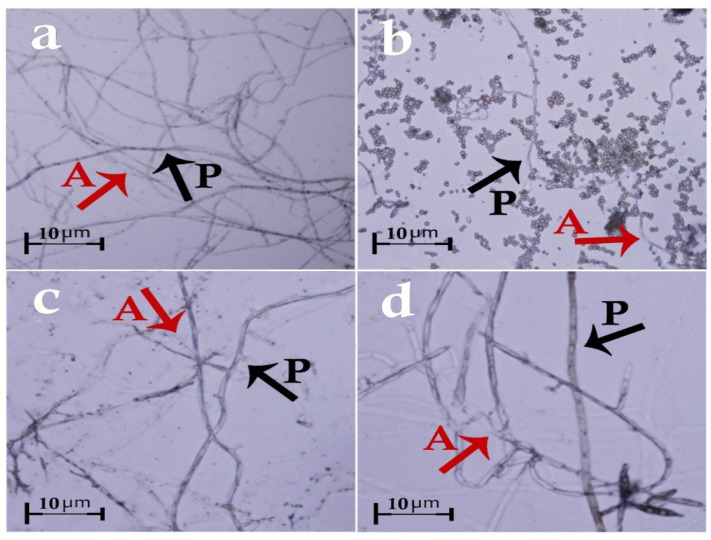
Hyperparasitism of *Rhizoctonia solani* by the studied antagonistic isolates (slide culture method): (**a**) *R. solani* × *Trichoderma virens*, (**b**) *R. solani* × *Aspergillus awamori*, (**c**) *R. solani* × *Aspergillus fumigatus*, (**d**) *R. solani* × *Curvularia lunata*. A: antagonistic fungi; P: pathogen.

It was found that the hyphae of *T. harzianum* and the pathogen did not reach each other, therefore, they had no interaction. On the other hand, the hyphae of *T. virens*, *A. awamori*, and *C. lunata* interfered with the pathogenic hyphae, connected to it, and cut the hyphae of *R. solani*. Moreover, the spores of *A. awamori* and *C. lunata* surrounded and deformed the pathogenic fungus’ hyphae. As for *A. fumigatus*, the mycelium reached, cut, and deformed the *R. solani* hyphae and caused its collapse (Figure 6).

### 3.4. Greenhouse Studies

#### 3.4.1. Evaluation of Disease Rating

**Figure 7:**
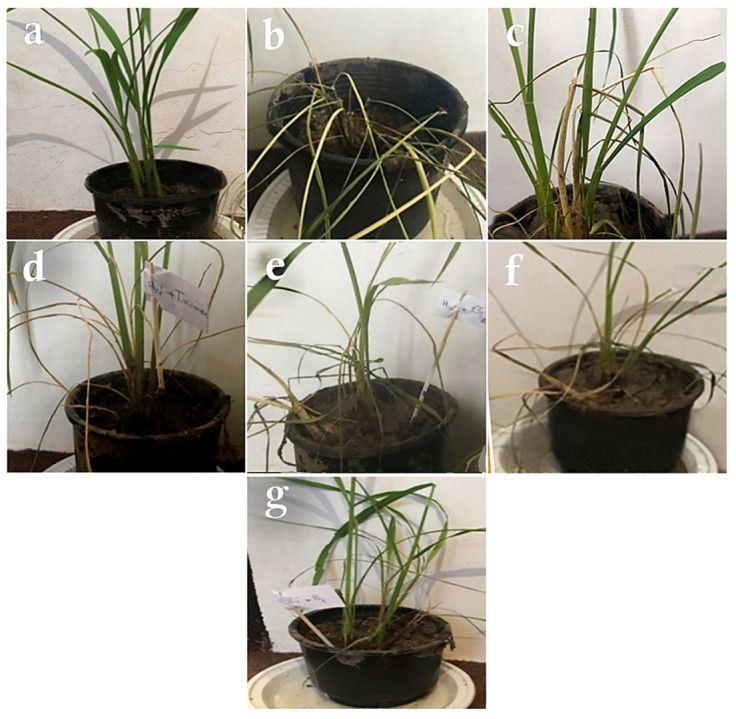
Greenhouse cultivation of rice plants: (**a**) plants treated with distilled water (negative control), (**b**) symptoms of the disease on rice after inoculation with *Rhizoctonia solani* only (positive control), (**c**) after inoculation with *R. solani* and *Trichoderma harzianum*, (**d**) after inoculation with *R. solani* and *Trichoderma virens*, (**e**) after inoculation with *R. solani* and *Curvularia lunata*, (**f**) after inoculation with *R. solani* and *Aspergillus fumigatus*, (**g**) after inoculation with *R. solani* and *Aspergillus awamori*.

No symptoms of the disease were observed in any of the control plants inoculated with distilled water (Figure 7a). As for the rice plants treated with *R. solani*, water-soaked spots that were gray or white in the center with a brown halo were the first symptoms that emerged three days after pathogen inoculation. The number of spots increased on the fifth day, and a complete blight of leaves was observed on the seventh day (Figure 7b). The mean severity of the disease was 10 in all the plants inoculated with *R. solani* only. As for the plants inoculated with *R. solani* and the antagonistic fungal isolates, the first disease symptoms appeared as gray to brown pinheads and white spots three days after inoculation. The infected spots appeared as greenish-gray, 1–3-cm-long, relatively irregular, oblong, and egg-shaped ovals. The center of the spots gradually turned grayish-white and their margins gradually turned brown (Figure 7c–g). 

Means of disease ratings of *R. solani* in the absence and presence of antagonistic fungal isolates are presented in Table 3. According to the results of the greenhouse trials, all of the studied isolates could reduce the severity of sheath blight disease. *T. virens* was found as the most effective by reducing the mean severity of *R. solani*-induced disease by 41.4%, followed by *A. fumigatus*, *T. harzianum*, *A. awamori*, and *C. lunata*, respectively (Table 3). 

The statistical analysis showed no differences in the disease rating index between the studied antagonistic fungi (5.86–7.30), although all of them reduced significantly the disease severity compared to the positive control (10.00) tested in the greenhouse. The highest reduction in the disease rating (41.4%) was found in the treatment with *T. virens*, while the lowest reduction (27%) was observed in the experimental object with *C. lunata* (Table 3).

#### 3.4.2. Evaluation of Plant Height

The results of the ANOVA showed that the antagonist fungi improved the longitudinal growth of the treated rice plants. The mean height of the rice plants inoculated with sterile distilled water was 55 cm (negative control). In the plants inoculated only with *R. solani* (positive control), an evident reduction in this parameter was observed, as the plants were ultimately 30 cm tall. As for the plants inoculated with *R. solani* and the five tested fungal isolates, the highest mean height was observed in the treatment with *A. fumigatus* or *T. harzianum*, or *T. virens* (47.00–50.66 cm) followed by *A. awamori*, and *C. lunata* treatments (37.33–38.00) (Table 3).

#### 3.4.3. Evaluation of Plant Fresh Weight

The mean fresh weight of the control plants inoculated with sterile distilled water was 12 g. In the plants inoculated with *R. solani* only, the mean fresh weight was reduced significantly and reached 7.30 g. Among the plants inoculated with *R. solani* and the five tested fungi, the highest mean fresh weight (9.16–10.00 g) was observed in the *T. virens*, *A. fumigatus*, and *T. harzianum* treatments, followed by *A. awamori* and *C. lunata* treatments. Generally, the antagonistic fungi increased the fresh weight of the treated plants, except for *C. lunata*, compared to those inoculated only with the pathogen (Table 3).

#### 3.4.4. Evaluation of Plant Dry Weight

The mean dry weight of rice plants inoculated with sterile distilled water was 2 g. As for the plants inoculated with *R. solani*, the mean dry weight was significantly lower (0.35 g). All of the tested antagonistic fungi were found to be effective in increasing the dry weight of the treated rice plants compared to the positive control. However, among the rice plants inoculated with *R. solani* and the five tested fungi, no significant differences in plant dry weight were reported—the value of this parameter reached from 0.61 g (with *C. lunata*) to 1.01 g (*T. virens*) (Table 3).

## 4. Discussions

*Rhizoctonia solani* is a widespread soil-borne pathogen that causes economically severe diseases in many crops, including rice. Currently, no rice cultivar completely resistant to this fungus has been identified [1]. Therefore, it is imperative to develop methods of plant protection alternative to chemical fungicides, which have drastic effects on the soil biota. Endophytes are microorganisms that reside within plant tissues and do not cause any deleterious effect on the plants. These microorganisms consist of bacterial and fungal communities that colonize and spend the whole or part of their life cycle inside the tissues of the host, without causing noticeable symptoms of plant diseases. Moreover, they can be useful in plant protection as natural antagonists of some fungal pathogens [36].

In the present study, 57 fungal isolates of *Rhizoctonia solani*, *Trichoderma virens*, *Trichoderma harzianum*, *Curvularia lunata*, *Aspergillus fumigatus*, and *Aspergillus awamori*, and some saprophytic fungi were collected from infected rice plants grown in the paddy fields in Guilan province, Iran. After a preliminary identification at the genus level, the isolates were subjected to pathogenic studies, and the pathogenicity of all of the *R. solani* isolates for rice plants was confirmed. Apart from the fungal isolates of *R. solani*-*T. virens*, *T. harzianum*, *C. lunata*, *A. fumigatus*, and *A. awamori* were selected for the biological control studies. 

In the dual culture method, the *T. virens* isolate was most effective in inhibiting the growth of *R. solani* (44.16% inhibition rate). Likewise, its inhibitory action reached 62.5% in the volatile metabolites assay, in which it was the most effective fungus for the mycelial growth inhibition of the pathogen along with *A. fumigatus*. This is consistent with the results obtained by Naeimi et al. [14], Abbas et al. [37] and Hanson [38] who reported the significant antagonistic effect of *T. virens* isolates against *R. solani* in zinnia and cotton plants. Fungi emit a large spectrum of biogenic volatile organic compounds (BVOCs) including acids, alcohols, aldehydes, aromatics, esters, heterocycles, ketones, thiols, and highly reactive terpenes [39]. According to Cook and Baker [7], due to the volatile secretions, isolates of *T. virens* were effective in reducing the growth of plant pathogens. Therefore, after 30 years, this endophyte can still be successfully used in the biocontrol of rice sheath blight disease. The volatile metabolites assay indicated higher values of growth inhibition in most experimental objects than the dual culture, which indicates the greater ability of the studied fungal isolates to produce volatile substances. This finding is consistent with the results of Safari Motlagh’s study [40].

In the hyperparasitism test, *T. harzianum* did not reach the *R. solani* hyphae, while the hyphae of *T. virens* reached and disintegrated the pathogen. This is inconsistent with the findings of Javadi et al. [41]. According to their research, no coiling of *Trichoderma* spp. (including *T. virens*) was observed around the hyphae of *Magnaporthe oryzae* (T.T. Hebert) M.E. Barr. On the other hand, microscopic studies conducted by Singh et al. [42] on the effect of *T. virens* isolates on *R. solani* showed that these isolates inhibited the pathogen’s growth and eventually destroyed it by interlacing, penetrating, and disintegrating its hyphae, which coincides with the present findings.

The five isolates that showed good in vitro antagonistic effects against *R. solani* also displayed good antagonistic activities in the greenhouse. *Trichoderma virens* was the most effective isolate in controlling rice sheath blight as it reduced the disease severity by 41.4%. *A. fumigatus* was another endophyte with great biocontrol potential (a reduction in the severity of rice sheath blight disease at the level of 36.7%). Moreover, *T. virens* increased the performance of rice plants (their fresh weight, dry weight, and height) infected with *R. solani*. According to Harman et al. [43], *Trichoderma* spp. can be a fungal pathogen parasite, which produces antibiotics that not only affect the pathogen but also have positive effects on plants, including increased germination, growth and yield, enhanced nutrients absorption, increased efficacy in the use of chemical fertilizers, and improved induced systemic resistance against plant diseases. This explains the present findings. 

Naeimi et al. [14] reported that among the *Trichoderma* species, *T. virens* is the most effective biocontrol agent for *R. solani*, which concurs with the present findings. However, they found that some of the endophytic strains prevented the production of *R. solani* sclerotia in vitro, although no relationships were observed between the biocontrol activity of these strains in vitro and their effectiveness in controlling rice sheath blight in the greenhouse, which does not agree with our findings. This inconsistency could be explained by Verma et al. [44], who reported that *Trichoderma* species are much more effective when applied as spores (especially conidiospores), which are resistant to unfavorable environmental conditions, than when used in mycelial forms or as propagules. For these reasons, a spore suspension was used in the present study. Future studies could focus on increasing the concentration of the spore suspension and/or changes in the method and time of its application.

Various species of the *Trichoderma* genus have been identified as biocontrol agents that can inhibit several plant diseases [45]. *Trichoderma* spp., which are common saprophytic fungi found in most soils and rhizosphere microflora, is known as a biocontrol agent owing to its ability to reduce the incidence of diseases caused by plant pathogenic fungi [46]. However, some of the *T. viride* strains have been registered as plant pathogens; e.g., in cucumber, pepper, and tomato; or antagonists of other plant-beneficial fungi, e.g., mycorrhiza-forming species [39]. The potential antagonistic mechanisms used by *Trichoderma* spp. include competition for habitat, nutrition, and antibiosis by synthesizing volatile and non-volatile compounds [14]. Therefore, careful screening for beneficial isolates is needed.

*Trichoderma harzianum* is another active component in many commercial biocontrol products [14]. In the present research, *T. harzianum* significantly reduced the disease severity and mycelial growth of *R. solani*. This endophyte was ranked the fourth most effective in controlling the pathogen’s growth in both dual culture and volatile metabolites methods and third in the greenhouse studies, which disagrees with the results obtained by Khodaee and Hemati [18] but corresponds with the findings of Matloob and Juber [17]. This inconsistency can be attributed to the differences in the genetic characteristics of the pathogenic and antagonistic fungi, the concentration of spores used for inoculation, and other factors, i.e., application method and time. Moreover, the biocontrol efficacy of *Trichoderma* isolates is reportedly affected by abiotic environmental parameters such as soil type, temperature, and water potential [47]. Toda et al. [48] reported that *T. harzianum* had the highest sheath blight control potential as it reduced the disease severity and increased the crop yield. The authors also revealed that spraying the plants with a suspension of fungal spores was the most effective way of applying this antagonist for the control of *R. solani*, while soil incorporation was the least effective method.

In the USA, biological control has been successfully used to reduce aflatoxin contamination in various crops, e.g., cotton, groundnut, and maize. This technique involves the soil application of a non-aflatoxigenic biological control strain of *A. flavus* or *A. parasiticus* that allows the antagonist to compete effectively with the native aflatoxigenic strains during invasion under favorable aflatoxin contamination conditions [49]. In the present study, *A. fumigatus* and *A. awamori* showed great in vitro and greenhouse biocontrol potentials against the pathogenic fungi. This finding concurs with the results obtained by Rosada et al. [50] and Cook and Baker [7]. Rosada et al. [50] identified isolates of *A. flavus* Link with good biocontrol potential against aflatoxin-producing agents in crops. In the present study, *A. fumigatus* ranked the second most effective in controlling the rice sheath blight fungal agent in the dual culture and greenhouse methods, and first in the volatile metabolite method. Likewise, Cook and Baker [7] found that *A. niger* had the greatest effect on preventing the linear growth of *R. solani*. In another study, Aybeke et al. [51] showed that *Aspergillus alliaceus* Thom and Church can be effective in the biocontrol of a root parasitic weed-*Orobanche* spp. Thus, *Aspergillus* spp. has good biocontrol potential against *R. solani* that could be utilized in Iran in the Guilan province and others where rice is planted. One should keep in mind, though, that *A. fumigatus* is a human pathogen that causes a fungal invasive disease called aspergillosis, which can be particularly dangerous in some groups of immunocompromised individuals [52]. Therefore, caution is advisable when using this fungus in crop protection.

## 5. Conclusions

Among the studied endophytic fungi, *T. virens* was the most effective antagonist in inhibiting the mycelial growth of *R. solani* in vitro. Likewise, in the greenhouse, it was the most effective isolate in reducing the severity of rice sheath blight. Therefore, *T. virens* was identified as the most effective biocontrol agent against this pathogenic fungus. *Aspergillus fumigatus* also showed good efficacy compared to the other antagonists, both in vitro and in the greenhouse, and as such promises the emergence of a new generation of biocontrol agents in the management of plant diseases. However, the fact that *A. fumigatus* is pathogenic to humans should be taken into account and the necessary tests need to be performed when using it as an antagonistic fungus.

Considering the limited number of sites from which the isolates were collected in this research, future studies are recommended to collect isolates from wider areas, such as paddy fields around the country. Moreover, other fungi species available in paddy fields should be used to find new antagonists. The next step would be to use fungi with proven antagonistic properties in the control of other plant pathogens, as well as bacterial antagonists.

## Figures and Tables

**Table 1:** Sequence of primers used in the PCR.

Name	Sequence (5′→3′)	TM	GC%	Mer
18s rDNA-F	CACCAGACTTGCCCTCCA	58.24	61.1	18
18s rDNA-R	AACCTGGTTGATCCTGCCAG	59.35	55	20

TM: melting temperature; GC%: GC-Content; Mer: number of nucleotides in each primer.

**Table 2:** Colonial growth and mycelium growth inhibition of *Rhizoctonia solani* by the studied fungi in the dual culture and volatile metabolites methods.

Fungal Isolates	Colonial Growth in the Treatments (mm)	Colonial Growth in the Control (mm)	Growth Inhibition (%)	Colonial Growth in the Treatments (mm)	Colonial Growth in the Control (mm)	Growth Inhibition (%)
	Dual Culture			Volatile Metabolites		
*Aspergillus awamori*	48.66 ± 0.21 a	78.00 ± 0.00 a	37.61 ± 0.01 a	42.00 ± 0.01 a	78.00 ± 0.00 a	46.00 ± 0.01 b
*Aspergillus fumigatus*	44.66 ± 0.02 a	78.00 ± 0.00 a	42.74 ± 0.01 a	25.00 ± 0.01 c	80.00 ± 0.01 a	68.75 ± 0.01 a
*Curvularia lunata*	46.00 ± 0.03 a	78.00 ± 0.00 a	41.02 ± 0.01 a	48.00 ± 0.01 a	78.00 ± 0.01 a	38.46 ± 0.01 c
*Trichoderma harzianum*	47.00 ± 0.01 a	78.00 ± 0.00 a	39.74 ± 0.01 a	43.00 ± 0.01 a	75.00 ± 0.01 a	42.66 ± 0.21 b
*Trichoderma virens*	43.55 ± 0.01 a	78.00 ± 0.00 a	44.16 ± 0.02 a	30.00 ± 0.01 b	80.00 ± 0.01 a	62.50 ± 0.01 a

Treatments having at least one similar letter did not show a significant difference according to the LSD test at *p* ≤ 0.01.

**Table 3:** Effect of antagonistic fungi on the sheath blight disease rating and the height, fresh weight, and dry weight of infected rice plants grown in the greenhouse.

Treatment	Disease Rating Index	Reduction in Disease Rating (%)		Plant Traits	
			Height (cm)	Fresh Weight (g)	Dry Weight (g)
Negative control	-	-	55.00 ± 0.02 a	12.00 ± 0.02 a	2.00 ± 0.02 a
Positive control	10.00 ± 0.00 a	0.00 ± 0.00 d	30.00 ± 0.03 d	7.30 ± 0.03 c	0.35 ± 0.03 c
*Aspergillus awamori*	6.97 ± 0.00 b	30.3 ± 0.03 c	38.00 ± 0.02 c	7.40 ± 0.03 c	0.73 ± 0.00 b
*Aspergillus fumigatus*	6.33 ± 0.00 b	36.7 ± 0.04 ab	47.66 ± 0.14 b	10.00 ± 0.06 b	0.96 ± 0.05 b
*Curvularia lunata*	7.30 ± 0.00 b	27.0 ± 0.04 cd	37.33 ± 0.13 c	5.90 ± 0.12 d	0.61 ± 0.00 b
*Trichoderma harzianum*	6.89 ± 0.00 b	31.1 ± 0.06 c	47.00 ± 0.00 b	9.16 ± 0.00 bc	0.92 ± 0.00 b
*Trichoderma virens*	5.86 ± 0.00 b	41.4 ± 0.05 a	50.66 ± 0.02 b	10.00 ± 0.02 b	1.01 ± 0.03 b

Treatments having at least one similar letter did not show a significant difference according to the LSD test at *p* ≤ 0.01. Negative control—plants inoculated with sterile distilled water. Positive control —plants inoculated with *R. solani* only.

## Data Availability

Data available by e-mail on reasonable request.
